# The affect of vision and compassion upon role factors in physician leadership

**DOI:** 10.3389/fpsyg.2015.00442

**Published:** 2015-05-08

**Authors:** Joann F. Quinn

**Affiliations:** Morsani College of Medicine, University of South FloridaTampa, FL, USA

**Keywords:** affect, role conflict, role endorsement, physician leadership, professionalism, identity

## Abstract

The career path for many professionals is often into a leadership role, yet many professionals do not have the competencies or inclination to lead. This study explores physician leaders as a representative group of professionals. While there have been many efforts at understanding the characteristics of effective physician leaders, a greater understanding is needed on the nature of physician leadership. The largest healthcare organization for physician leaders in the United States was surveyed to gain a greater understanding of the nature of leadership. Partial Lease Squares (PLS) was used to analyze results from 677 online surveys to understand the causal relationship of role conflict and role endorsement to participation. The findings reveal the mediating influence that positivity exerts upon participation, and offers health care leaders an opportunity to increase understanding of the social identification process that leads a higher level of professional participation, which may increase effectiveness for physicians in leadership.

## Introduction

Physicians have long held leadership roles within hospitals and other healthcare organizations (Reinertsen, 1998). While there is considerable research on physician leadership from a variety of disciplines (Lobas, 2006; Chaudry et al., 2008), there is a lack of focus on the *nature* of physician leadership. This paper proposes that a key factor in how physicians understand their leadership roles is tied to the construction of a leadership identity, which shapes their understanding and enactment of their role as a leader. Identity is an ongoing process of framing, based upon interactions with others and the environment (Foreman and Parent, 2008).

The differentiation in identity from a clinician to a leader offers a unique perspective of study that could greatly impact how we understand physician leadership. The challenge for healthcare organizations concerned with improving physician leadership goes beyond selection and development (Stoller, 2009), with a greater focus needed on how physicians understand and construe their roles as leaders. As a unique group of highly educated, professionals that place such a high value in their individual role as clinicians, the nature of physician leadership is shaped by how they embrace and understand their role as a leader. The findings of this study may also be applicable to other professional services leaders who are part time and/or temporary, while they retain their professional role as an individual contributor.

To better understand the nature of physician leadership, this study seeks an understanding of the impact of role conflict, as well as role endorsement, upon physician leader participation. It offers a model, which theorizes that two aspects of positive affect (compassion and vision) mediate the relationship of role conflict and role endorsement upon participation and seeks to validate these hypotheses based upon responses from 677 physician members of the American College of Physician Executives.

The understanding of the nature of physician leadership is an important topic to explore as it has implications at both the practitioner and theoretical level. While many theories of leadership exist, none specifically have sought to understand leadership from the perspective of an at times unwitting physician leader who is thrust into the role, which may be temporary and even part time.

This paper begins with a review of the literature on physician leadership, identity and role, positive affect and organizational participation. Building upon existing theory, a theoretical framework is developed, along with an associated set of hypotheses. The research methodology and sample are then presented, along with the analysis of results, as well as a discussion of findings. Finally, the paper concludes with a discussion of the implications to research and practice.

## Theoretical foundation

In this section, literature discussing physician leadership, the impact of positive relationships, identity and role will be reviewed.

### Physician leadership

Like many other professionals, physicians often assume part or full time leadership roles as department chairs, committee members, directors, and other administrative roles in hospitals and healthcare organizations. These leadership roles are often held within clinical departments or specific functions that operate somewhat separately from the larger organization (Lobas, 2006). There is a need for a higher level of involvement from a managerial perspective because of increasing pressure from the way medicine is “*determined,” “accessed,” “organized,” “monitored,” “delivered,”* and “*paid for,”—*and this need is being placed upon physician leaders (Montgometry, 2001).

Montgomery suggests that the intra-professional divisions between clinicians into areas of functional expertise may not be as relevant as the division of physicians into clinician and manager due to the changing structure of healthcare (Montgometry, 2001). This separation between clinical roles and physician leadership roles starts to look like two different professional groups. In addition, several physicians interviewed in this author's prior exploration cited excellent physician leaders as brilliant surgeons or outstanding researchers, not realizing the absence of including leadership competencies in their criteria for excellence. The connotation of leadership then seems to be understood by the physician as individual expertise and contributions rather leadership competencies. As far back as the fourth century BC, Plato's teachings inform us that the ideal leader is someone who commits himself to fellow citizens (Plato). The nature of physician leadership, therefore, may be explained by the focus of the profession being the physical wellbeing of their fellow citizens.

How then, can physician leaders be developed into successful organizational leaders? Physicians are educated and professionalized to value autonomy (Stoller, 2009; Blumenthal et al., 2012), yet in order to succeed as an organizational leader, collaboration and leadership competencies are necessary.

The increasingly complex environment in healthcare also requires more inter-departmental collaboration VanVactor (2012). Yet, Hall (2005) notes that there is an inherent challenge for members of different professional groups to collaborate, as they have different cognitive maps, which develop as a condition of their professionalism. Physician leaders hold a unique opportunity to serve as boundary-spanners, because they speak the language of and relate to both administration and clinicians (Sherrill, 2000). In order to bridge the gap between administration and medical staff, physician leaders must face the “tribal stigma” (Goffman, 1963) that exists between members of different social groups.

### Affect

**Figure 1:**
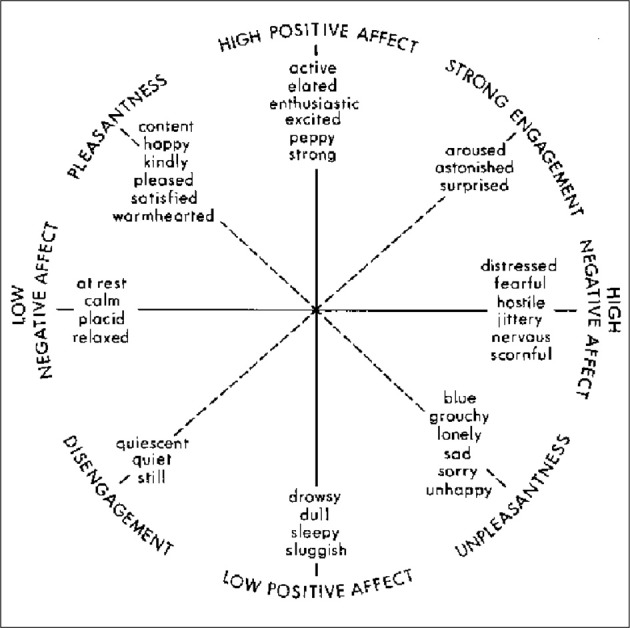
**Watson and Tellegen's (1985) two-dimensional map**.

The field of positive psychology has aided in our understanding of what leads to individuals thriving emotionally at the individual, community and societal level (Seligman and Csikszentmihalyi, 2000). In this paper, we are particularly concerned with the role of affect, which Tellegen et al. (1999) define as positive and negative emotional activation. In an effort to define affect, Watson and Tellegen (1985) constructed a model with a two-dimensional structure that plots high and low positive and negative affect, as well as two other dimensions of un/pleasantness and dis/engagement. See Figure 1 for a representation of this model. It is an important consideration that “positive affect” and “negative affect” are separate dimensions- not at opposite ends of the same continuum. Psychologists have found evidence that positive affect is not the bipolar opposite of negative affect: “it seems that a human being is not a pendulum, moving between opposite feelings” (Russell and Carroll, 1999, p. 3), rather an individual can have feelings of happiness and sadness at the same time.

Boyatzis has argued that Positive Emotional Attractors (PEA) and Negative Emotional Attractors (NEA) are critical in affecting behavior, influencing one on a cognitive, emotional, social and physiological basis (2008). Positive and negative emotional attractors are described by Boyatzis (2008) as destabilizing forces that create psycho-physiological states that drive the change process. These “strange attractors,” first introduced by Lorenz (1963), “create forces that pull our behavior, attitudes, and feelings around them, but not into them (Boyatzis, 2008). According to Boyatzis (2006), experiences in the Positive Emotional Attractor (PEA) are thought to arouse neuro-endocrine systems, and this stimulation leads to higher cognitive functioning, increased openness to ideas, emotions and people, positive emotional states, and increased immune health. Conversely, the stimulation of the Negative Emotional Attractor (NEA) leads to a decrease in cognitive function, perception and immune function (Boyatzis, 2006). As noted in the Watson and Tellegen model, the PEA and NEA model is grounded in the theory that positive and negative affect are not merely bi-polar (Russell and Carroll, 1999), but instead are represented by three different dimensions (Boyatzis, 2008). Jack et al. (2013) demonstrated the neural response to coaching to either the PEA or NEA; the PEA coaching activated the parasympathetic nervous system (PNS), while the NEA activated the sympathetic nervous system (SNS). Their results show the relationship of positive affect to behavior is a physiological one, not solely based in theory. The key is to have the proper ratio of the negative and positive attractor for effective functioning.

The PEA is related to a shared vision, compassion and overall positive mood (Boyatzis and McKee, 2005), and refers to the relationship of the leader and the followers, such that both the leader and follower are affected, and it has been shown to impact the follower's job satisfaction, organizational commitment, turnover intention, health, effort, learning, and development (Bass, 1990; Gerstner and Day, 1997; Bommer et al., 2004). Shared vision is the process in which the PEA moves between people. The perception of shared vision, one of the sub-scales of the P/NEA survey (Boyatzis, 2008), has been shown to predict championing behavior (Clayton, 2009), success in family businesses (Neff, 2011), and even increased organizational engagement (Mahon, 2010).

Compassion, another sub-dimension of Boyatzis' P/NEA measure (Boyatzis, 2008) plays a fundamental role in our human existence, and is vital to our humanity (Himmelfarb, 2001). Boyatzis (2011) define compassion as “an interpersonal process that involves noticing another person as being in need, empathizing with them and acting to enhance their well-being in response to that need.” While compassion has been studied for thousands of years within philosophy, sociology and religion, its value has often been overlooked within organizations (Kanov et al., 2004). Compassion is essential as a connection between individuals within an organization (Frost et al., 2000). Just as compassion is an element in ideal physician-patient relationships (Rayburn, 2006), physicians who take on leadership roles must also value the importance of compassion in relationships with the other physicians they work with and lead.

Therefore, the following hypotheses are proposed:

*Hypothesis 1. Shared vision has a positive effect upon an increased level of participation in leadership activities*.
*Hypothesis 2. Compassion has a positive effect upon an increased level of participation in leadership activities*.
*Hypothesis 3. Mood has a positive effect upon an increased level of participation in leadership activities*.

### Identity, professionalism, and role

Stets and Burke (2000) demonstrated that aspects of identity theory and social identity theory can provide a more comprehensive view of the self than either individual theory; therefore we should look to *both* theories to understand the social construction of identity for physician leaders. Central to identity is the self within the role, as well as the meanings associated with that role Burke and Tully (1977). The individual's identity is formed by the reflexive self-categorization and identification as a member of a group or role (Stets and Burke, 2000). The impact of professionalism is also explored, which is so distinct for physicians beginning with their white coat ceremony in medical school and continuing throughout their career, and how these influence role factors of role conflict and role endorsement.

Individuals define their identity through membership within various groups, such as work groups, organizations (Tajfel and Turner, 1985) and as members of a profession (Tajfel and Turner, 1985; Ashforth and Mael, 1989), and social settings determine the characteristics of people likely to be in that environment (Goffman, 1963). Physicians' social identification then places them (and others) into categories of classification within their environment and separates themselves as physicians from certain “others” in the organization.

According to Larson, the focus upon the uniqueness and specialization of the role exaggerates the “dignity” of the profession (Larson, 1979, p. 490), forming the professional self. The individual, in this case the physician, adopts an identity focused on the primary function (as a clinician), which is given a superior priority and distinction. Larson observes that professionals are “locked in by their vocational choice, by the particular mystique of each profession, and by their whole sense of social identity” (Larson, 1979, p. 490). While physicians, managers, and administrators are all members of the greater organization and health care community, each sees themselves as in terms of their profession, which “confines the professional” to that primary identity (Larson, 1979).

This social classification process begins in medical school. Physicians are not only gaining technical expertise, but are also being socialized into a profession and assuming their identity as a physician (Hall, 2005). Individuals assign themselves to a classification for emotional value (Tajfel, 1974) which is predicated on the respect that they receive (Ashforth and Mael, 1989). When they then shift into roles of physician-leadership, the majority hold on to their primary identity of physician (Montgometry, 2001). The “value” of their identity lies in their expertise and education as a physician, which has been reinforced through their professional group.

Social identity theory explains that the process of self-categorization accentuates the similarities of those belonging to the same category and the differences of those in different categories (Turner, 1985). Thus, people are depersonalized and construed as in-group and out-group members (Hogg et al., 1995). As physicians adopt a universal persona, depersonalization is *not a negation* of identity. Instead, the individual changes the perception of his/her identity to that of the group he/she identifies with (Hogg et al., 1995). As a result of self-categorization, individuals create prototypes to represent social groups. These prototypes are defined by the greatest similarities between group members, focusing on the positive attributes of members, as well as the differences that set the group apart from others (Hogg and Terry, 2000).

Not only are physicians then confined to a professional group that excludes others, but there is reluctance to become subordinate to those outside of their group (Bate, 2000). This may even extend to their view of their own peers in leadership roles. As physicians accept leadership roles, they are expected to support organizational goals, which may be different than their own goals as clinicians, as well as those of their clinical colleagues. As a result of their professionalization, physicians themselves impact the organization by influencing the perception of the roles—including those of physician leaders—within it. Professionalism does not only impact the understanding of the role for an individual, it affects how the role influences identity. Holden et al. (2012) suggest that professional identity formation is a series of processes that includes professionalism, psychosocial identity development and formation, which transforms the individual from a layperson to physician. An understanding of how an individual understand their own identity is important in assessing the impact of role factors upon enacted behavior.

Social identity theory aids our understanding of the nature of physician leadership with respect to the multiple roles they must assimilate, which does not specifically address “roles,” but does set out “to explain individuals' role-related behaviors” (Hogg et al., 1995). Through a series of reflexive social interactions, individuals acquire meaning; thus clarifying their own roles as well as the roles of others (Burke and Reitzes, 1981). A leadership role is conceptualized by the individual in response to the expectations of others (Boyatzis, 1982), and as physicians adopt their own role identity, they interact with other physicians, nurses, administrators and professionals within the organization, developing self-meaning and definition through their actions and the social structure.

The role of physician, or leader, then creates a norm for behavior as an incumbent of that role, and in turn “the self as a structure of role-identities… operate[s] as a social force, affecting the structure of society by affecting behavior in important ways” (Callero, 1985, p. 203, citing Rosenberg, 1981). The self is now considered to be “multiple, varied, changeable” and in fact may adapt to the context (Salgado and Hermans, 2005, p. 3). Thus, the construction of meaning for an individual is dependent upon relationships with other people, including an individual's meaning of self (Cross et al., 2000; Salgado and Hermans, 2005).

**Figure 2:**
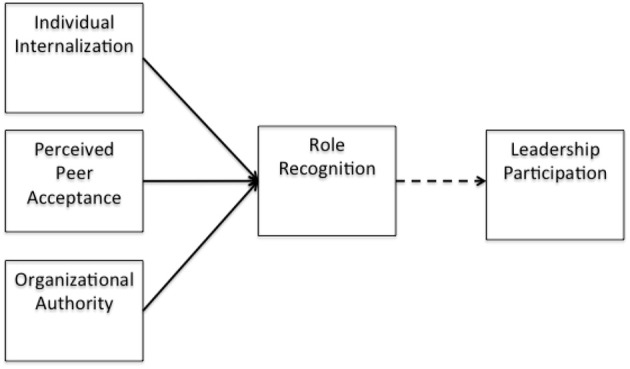
**Role recognition**.

DeRue and Ashford (2010) proposed that, “if a person claims leadership in a setting but others do not reinforce that claim with supportive grants… leadership identity construction (is) insufficient for a leader-follower relationship to emerge,” therefore it is expected that physician leaders who hold part time leadership roles, as well as full time clinical roles relate to physician managers as “tribe” members, and often not as leaders. However, the sub-constructs of the positive emotional attractor: vision, compassion and mood, may encourage a more flexible outlook for the individual to adapt their understanding of their role as a physician leader and embrace their secondary identity to engage more as a leader. See Figure 2 for a representation of role endorsement.

*Hypothesis 4. Vision partially mediates the positive relationship of role endorsement upon participation*.
*Hypothesis 5. Compassion partially mediates the positive relationship of role endorsement upon participation*.
*Hypothesis 6. Mood partially mediates the positive relationship of role endorsement upon participation*.

Moreover, identity theory informs us that that role-identities are hierarchically positioned, thus having differing effects upon behavior (Callero, 1985; Hogg et al., 1995). Therefore, even when an individual accepts additional identities, there is still a primary identity that may inform behavior. This may be the source of role conflict for physicians.

“According to the chain-of-command principle, organizations set up on the basis of hierarchical relationships with a clear and single flow of authority from the top to the bottom should be more satisfying to members and should result in more effective economic performance and goal achievement than organizations set up without such an authority flow” (Rizzo et al., 1970). This idea was established decades ago, yet the premise still remains. Role theory offers that individuals become stressed and dissatisfied when the behaviors expected in their role are inconsistent (Rizzo et al., 1970). Role conflict therefore occurs when the expectations of the role are contradictory (Hardy and Conway, 1978). The very structure of some hospital and healthcare organizations may then create role conflict.

Increasing the level of participation and improving performance is in effect dependent upon a greater understanding of role acceptance and conflict. While many scholars have studied role conflict (Kahn et al., 1964; Rizzo et al., 1970; House and Rizzo, 1972; Jackson and Schuler, 1985; Friedman and Podolny, 1992), questions still remain as to what the impact of role conflict is, and how to measure the consequences.

Negative emotions, such as those associated with role conflict, are correlated with a lower likelihood of cooperation (Cremer and Hiel, 2006). Yet, job satisfaction has been found to mediate role stressors to organizational citizenship behavior. In this analysis, the impact of role conflict is of interest as one form of role stressor, and its affect upon participation, which is a sub construct of organizational citizenship behavior. Therefore, the following hypotheses are explored:

*Hypothesis 7. Vision partially and positively mediates the negative relationship of role conflict upon participation so that the relationship of compassion to participation is positive*.
*Hypothesis 8. Compassion partially and positively mediates the negative relationship of role conflict upon participation so that the relationship of compassion to participation is positive*.
*Hypothesis 9. Mood partially and positively mediates the negative relationship of role conflict upon participation so that the relationship of compassion to participation is positive*.

## Methodology

To explore the nature of physician leadership, a survey-based study was conducted to validate the hypotheses. A psychometric survey methodology was used that maps individual responses to the underlying concepts within the model. In an effort to capture representative data on physician in leadership, the membership of the American College of Physician Executives (ACPE) was surveyed.

### Measurement of research variables

To ascertain and measure the relevant dimensions of the model, this process proceeded in four stages: development of the survey instrument, development of measurement scales, pretesting to assess validities of the survey instrument and data collection from a sample of physicians with membership in the American College of Physician Executives (ACPE), the largest health care organization for physician executives in the US.

Where possible, construct items were based upon previously validated measures; otherwise, indigenous items were developed based on a review of pertinent literature and using a procedure consistent with prior studies (Churchill, 1979; Koufteros, 1999). All first-order constructs were specified with reflective indicators, except for Participation. Participation is defined by five formative indicators adapted from the work of Van Dyne et al. measure of organizational citizenship behavior Van Dyne et al. (1994), with the belief that these indicators cause participation.

### Construct development

Although most scale items were adapted from those in the existing literature with slight modifications to reflect the focus of this study, a new scale was developed to measure role endorsement.

#### Independent variables: role conflict and role endorsement

Role conflict items were adapted from the work of Rizzo et al. (1970). The scale contained 15 items, which were measured on a 5 point scale ranging from 1−“Strongly Disagree” to 5−“Strongly Agree.” Four role conflict items were selected that were believed to be most suited to this inquiry.

Role endorsement was informed by the author's earlier work on physician leadership and adapted from DeRue and Ashford (2010). Six items were developed to measure the claiming and granting of leadership within peer relationships, as well as from an organizational perspective.

#### Dependent variable: participation

A multi-item construct of organizational citizenship behavior was adapted using participation as a major component. The five measures of participation were adopted from the original 54 items in Van Dyne et al.'s measure for organizational citizenship behavior Van Dyne et al. (1994). These items were measured on a 5-point scale ranging from “1-strongly disagree” to “5-strongly agree.”

#### Mediating variable: positive emotional attractor

To measure positivity, Boyatzis' PNEA scale (2008) was used, which includes three subscales, vision, compassion and overall positive mood. All items were measured on a 5-point scale with “strongly agree” at the extreme positive end and “strongly disagree” at the opposite end of each scale.

#### Controls

Several controls were also included, including role, tenure in role and in organization, age and gender. Rousseau and McLean Parks (1993) noted that employees who have long tenure in their organizations tend to have strong organizational ties, and it has been found that the confidence developed in a role leads to increased competence and feelings of organizational commitment (Salancik, 1977). Age and gender have also been used in numerous studies across disciplines to assess impact upon results, and these were included to ensure the accuracy of the data.

**Figure 3:**
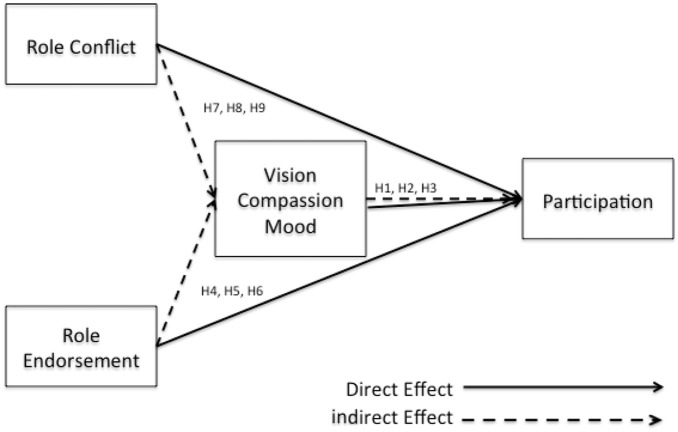
**Conceptual model**.

The multi-items for each of the constructs are summarized in Appendix A and the relationships of the model represented in Figure 3.

### Sample

The population sampled was the membership of the American College of Physician Executives (ACPE)^*^, which is the largest organization for physician executives in the nation. The ACPE is accredited by the Accreditation Council for Continuing Medical Education and has greater than 9000 members from the United States and 45 other countries, holding roles including chief medical officer, chief executive officer, vice president of medical affairs, directorships, as well as others (ACPE Website).

The survey was delivered online, which was emailed out to members of the American College of Physician Executives (ACPE) by the management of the organization. The ACPE has over 9000 registered members who are self-selecting into the organization, with the requirement of full members being allopathic (MD) and osteopathic (DO) physicians; dentists (DDS or DMS); and podiatrists (DPM).

Individual respondents were provided a URL to the survey, which was deployed through Qualtrics, a popular online survey research tool. Of the 9083 contacts that received the email with the survey link, 8672 emails were delivered, 2148 were tracked as opened, 1030 clicked on the link for the survey, and 936 physicians started the survey. The sample was then reviewed for missing values resulted in a final sample size of 677.

The data was collected beginning in July 2011, with 547 males and 128 females responding (81 and 19%, respectively). Of the respondents, 420 stated their leadership role as part time and 222 as full time, with the remaining responding with “not applicable.” 308 (46%) of respondents stated their age as 55 or older and of that age group, 240 reported their role as full time. The American Medical Association (AMA) delegates reported 79.4% as male and 20.6% as female and 77.3% as over age 50 as of December, 2010 (AMA, 2011, Annual Meeting), suggesting that this sample is objectively representative of the population of physicians in leadership, as well as the surveyed population.

### Measurement and instrument development

In developing the survey instrument, a list of itemized questions was sent to 10 respondents, including several physician leaders, and asked them to comment on the flow, clarity, timing, and the respondents' interest through completion rate. Two of the items were modified to ensure that exact meaning was conveyed and understood. The pre-test was then followed by asking three individuals to read the questions aloud and answer them in order to assess cognitive difficulties presented by the survey items (Bolton, 1993). The language used in one item was also adjusted for clarification.

Next, a pilot survey was conducted with 65 physicians working in four hospitals within a single healthcare organization to perform an exploratory factor analysis (EFA) for each hypothesized construct within the model. The pilot survey was carried out online. The items were found to be acceptable for factoring within each construct and no adjustments were made following this step.

### Data screening

Prior to analysis, missing values were removed related to the latent constructs. The data was screened for linearity, normality, multicollinearity, skewness, and outliers and found the data adequate for analysis. 260 data points were dropped due to missing values. There were no significant outliers, as the survey contained primarily Likert scales.

### Statistical analysis

The research model was tested using Partial Lease Squares (PLS-Graph, v3.0, Build 1060, Chin and Frye, 1998). An assumption for a covariance based SEM analysis is that the items used to measure a latent variable are reflective (Chin, 2010). “Since PLS explicitly estimates the outer weights to form construct scores, modeling formative indicators is much less problematic” (Chin, 2010, p. 664). Jarvis et al. (2003) provide a set of four decision rules based on: (1) direction of causality based on conceptual definitions, (2) interchangeability of the indicators, (3) co-variation among the indicators, and (4) nomological net of the indicators. Taken together, these rules can suggest either a reflective or formative model formulation. It was found that the dependent variable, participation, is formative in nature, therefore the use of PLS-Graph was necessary to analyze the model.

### Measurement model

#### Exploratory factor analysis- reflective constructs only

An EFA (exploratory factor analysis) was performed using principal axis factoring and PROMAX rotation. Sample size was adequate with 677 usable responses across 28 items. The Kaiser-Meyer-Olkin (KMO) value was 0.932 and the Barlett's Test of Sphericity was significant (*x*^2^ = 8935.759, *df* = 378, and *p* < 0.000), indicating sufficient intercorrelations for factors to emerge. The analysis was run initially by selecting factors with Eigen Values over 1. Using this criterion, five latent constructs hypothesized *a priori* in the model, emerged from the data. The constructs explained a little over 48% of the variance within the data. A sensitivity analysis was also conducted by re-running the EFA specifying 6 and 7 factors, but found considerable cross-loadings, in particular mood loaded across three factors, with both negative and positive results. As this was contrary to theory, mood was eliminated from the final model.

The pattern matrix for initial convergent and discriminant validity. Criterion was employed as designated by Hair et al. (2011), which states that factor loadings in the range of 0.3 to 0.4 are considered acceptable for interpretation of structure, and given the sample size of 677, each loading over 0.3 is considered statistically significant. The criteria used by Igbaria et al. (1995) was used to identify and interpret factors which were: each item should load 0.50 or greater on one factor and 0.35 or lower on the other factors. It was found that one item in the role endorsement construct was a bit low and had one cross loading. Yet, the cross loading was greater than 0.2 and therefore the item was retained into the confirmatory factor analysis (CFA). See **Table 6** for full results of the confirmatory factor analysis.

After eliminating one item (role endorsement item 1), 27 items measured five factors—four reflective and one formative. Table 1 shows the reliability of each of the four reflective factors. Table 2 provides the correlations between factors. The EFA results provided the foundation for further testing using PLS-Graph (v3.0, Build 1130, Chin and Frye, 1998).

**Table 1:** **EFA measurement model: reflective constructs**.

**Construct**	**Number of items**	**Loadings**	**Cronbach's alpha**
Role conflict	4	−0.736, −0.652, −0.591, −0.541	0.728
Role endorsement	5	0.866, 0.854, 0.721, 0.695, 0.464	0.872
Vision	8	0.876, 0.853, 0.818, 0.805, 0.689, 0.604, 0.590, 0.586	0.912
Compassion	6	0.773, 0.767, 0.656, 0.620, 0.518, 0.437	0.841

**Table 2:** **Correlations**.

	**Role conflict**	**Role endorsement**	**Vision**	**Compassion**	**Participation**
Role conflict	1				
Role endorsement	−0.414	1			
Vision	−0.433	0.644	1		
Compassion	−0.389	0.568	0.642	1	
Participation	−0.111	0.357	0.289	0.291	1

Partial Least Squares (PLS), a structural equation modeling (SEM) technique, was used for testing the research model. PLS approach was superior to other SEM approaches for this study because of its flexibility on distributional assumptions, its small sample size requirements, and its strength on complex predictive models (Chin and Newsted, 1999). PLS is a regression-based technique with roots in path analysis (Fornell and Larcker, 1981; Chin and Frye, 1998); however, it has emerged as a powerful approach to studying causal models involving multiple constructs with multiple indicators. This approach facilitates testing of the measurement model and the structural model simultaneously. The measurement model revalidated the instrument and determined how each manifest variable's loaded on the construct that it measured. The structural model was estimated using the PLS algorithm with bootstrapping (1000 resamples).

### Assessment of the measurement model

To assess the psychometric properties of the latent constructs, a PLS measurement model was created. To assess convergent validity, the internal consistency reliability (ICR), the average variance extracted (AVE), and the item factor loadings for the reflective constructs were assessed.

#### Estimation of internal consistency

The survey employed multi-item scales to measure the reflective first-order factors. The measurement properties for the reflective constructs were examined by conducting confirmatory factor analyses using PLS. To assess the internal consistency of the reflective factors, AVEs, coefficient alpha and composite reliability measures were assessed. For participation, it was not possible to assess validity and reliability, since the very nature of formative measurement renders irrelevant traditional assessments of convergent validity and item reliability.

Accordingly, as seen in **Table 4**, coefficient alpha values ranged from 0.786 to 0.887. Likewise, the composite reliabilities for all reflective measures were high, ranging from 0.831 to 0.929. The recommended level for establishing a tolerable reliability is the 0.70 threshold. All reflective construct coefficients were above 0.831 showing strong reliability.

Tests were conducted to evaluate the convergent and discriminant validity and the reliability of reflective measures. Convergent validity of the constructs is assessed by examining the constructs factor loadings, composite scale reliability and average variance extracted (Fornell and Larcker, 1981; Chin and Frye, 1998). Loadings in excess of 0.70 on their respective factors are interpreted to indicate convergent validity (Straub et al., 2004). A second indicator of convergence was also employed. Here, a value above 0.50 for the average variance extracted (AVE) for each construct is assumed to indicate sufficient convergence. As seen in Table 2, results indicate that both of these conditions have been met. Discriminant validity is demonstrated when the square root of the AVE is greater than the correlations between constructs (Bollen, 1989). Note that although two items within the participation construct were found to be not significant (PART 3 and PART 4), the items were retained, as removal would alter the nature of that formative construct.

The square root of AVEs ranged from 0.553 to 0.663 for reflective constructs. For a second test of discriminant validity, individual items may be assumed to possess sufficient discriminant validity if they load higher on their own respective construct than on any other latent variable (Gefen et al., 2000; Straub et al., 2004). This was true for all items. Based on both tests, the measures possess sufficient discriminant validity. Consequently, evidence for internal consistency and the scales reliability were provided by the results, which can be found in the appendix.

### Dimensionality and convergent and discriminant validity

It was expected that items belonging to the same scale would have factor loadings exceeding 0.70 on this common factor. As indicated by the results in **Table 6**, although all the loadings were statistically significant based on *t*-statistics generated from running a bootstrap on the data, none were below the acceptable threshold (0.60). Moreover, the average variance explained (AVE) was below 0.50 and considered unacceptable.

As a result of the construction of a formative variable, “conventional procedures used to assess the validity and reliability of scales composed of reflective indicators (e.g., factor analysis and assessment of internal consistency) are not appropriate for composite variables (i.e., indexes) with formative indicators” (Diamantopoulos and Winklhofer, 2001). One of those measures that is not appropriate for formative constructs is AVE, which is the measure of the amount of variance that indicators provide to the latent variable, relative to the measurement error. For those reflective constructs, AVE should be 0.50 or greater, which explains 50% or more of the variance (Chin, 2010). The composite reliability (CR) for each construct is found in Table 6 (below). The CR for each reflective construct exceeds the acceptable threshold (>0.70) and the average variance extracted (AVE) confirms the reliability of the indicators and demonstrates convergent validity.

### Common method bias

A test for common method bias was performed, as survey item responses were all self-reported. In order to test for common method bias, Harman's one-factor test was applied, including all items in the model in a principle components factor analysis (Podsakoff et al., 2003). If one factor accounts for the majority of the covariance, common method bias is present. Based upon Eigenvalues greater than 1, 5 factors emerged, which explained 48% of the variance, therefore it appears that there is no common method bias. The correlations matrix was also examined, as common method bias can also be assessed from these values. Correlations above 0.90 are indicative of a common method bias problem (Pavlou et al., 2007). No correlations were found to be near the 0.90 level, which suggests that there is no evidence of common method bias.

### Structural model

The test of the structural model includes estimating the path coefficients and the *R*^2^-values. The path coefficients, which indicate the strength and direction of the relationships among the variables, should be significant and directionally consistent with expectations. The R^2^, which represents the proportion of variance in the endogenous variables that can be explained by the antecedents, demonstrates the predictive power of the model. Collectively, R^2^ and path coefficients indicate how well the model fits the empirical data (see Table 3 for effect sizes). To assess whether the main effects were significant, bootstrap resampling was performed. Bootstrapping (677 resamples) was used to create sub-samples from which the *t*-values associated with various inner and outer model paths in the model were obtained (Chin, 2000).

**Table 3:** **Path analysis, hypotheses and effect sizes**.

**Hypothesized relationship**	***R*2**	***t*-Statistic**	***f*^2^**	**Strength**
H7: Role conflict → Vision	0.448	6.498	0.0598	Small effect
H4: Role endorsement → Vision	0.448	19.0748	0.4710	Large effect
H7: Role conflict → Compassion	0.348	4.7257	0.0414	Small effect
H5: Role endorsement → Compassion	0.348	14.4921	0.3037	Medium effect
H1: Vision → Participation	0.107	2.2769	0.0134	No effect
H2: Compassion → Participation	0.107	3.444	0.0269	Small effect

A series of tests were run to investigate the predictive power of the structural model (Chin and Frye, 1998). The model was tested for the change in R^2^, to determine the substantive impact of each independent variable upon the dependent variables. To do so, f^2^ was calculated in the following manner:

$$f^{2}=\frac{R^{2}\;included-R^{2}\;excluded}{1-R^{2}\;included}$$

**Figure 4:**
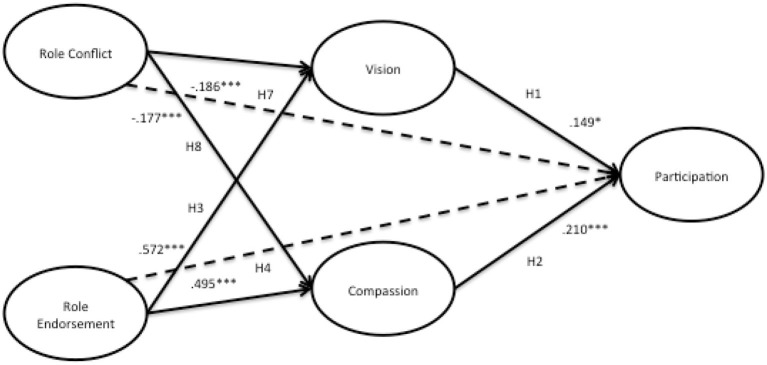
**Structural model**. ^*^p < 0.05; ^***^p < 0.001.

R^2^ represents the amount of variance in the construct that is explained by the model. Cohen (1988) recommends values of 0.02, 0.15, and 0.35 to denote small, medium, or large effects at the structural level. The causal four-steps method developed by Baron and Kenny (1986) was used to test for mediation effects, presented in Table 4, below. Analysis of our structural model revealed four mediated relationships where a significant independent variable (IV)—dependent variable (DV) relationship was mediated. Please see Figure 4 for the structural model.

**Table 4:** **Mediation results of compassion and vision**.

**Mediated path**	**Path coefficient**	***t*-Statistic**	**StdErr**	**Effect**
H4: RE → P	0.364^***^	8.2065	0.0452	Partial mediation
RE → VI	0.572^***^	19.2890	0.0297	
VI → P	0.149^*^	2.2789	0.0654	
H5: RE → P	0.364^***^	8.2065	0.0452	Partial mediation
RE → COMP	0.495^***^	14.3085	0.0346	
COMP → P	0.210^***^	3.5177	0.0597	
H7: RC → P	0.033*NS*	0.2773	0.0433	Full mediation
RC → VI	−0.186^***^	6.2113	0.0382	
VI → P	0.149^*^	2.2789	0.0654	
H8: RC → P	0.033*NS*	0.2773	0.0433	Full mediation
RC → COMP	−0.177^***^	4.6366	0.0382	
COMP → P	0.210^***^	3.5177	0.0597	

* *p < 0.05*;

^**^p < 0.01;

*** *p < 0.001*.

## Findings

See Table 5 for the effect size of each relationship. Although the amount of variance explained by Participation is low (*R*^2^ = 0.107), it should be noted that this is an exploratory model. All other effects are within the appropriate range (Cohen, 1988). The hypothesized structural model was tested in PLS and the results showed two negative and four positive relationships between constructs.

**Table 5:** **Results**.

**Hypothesis**	**Result**
*H1: Shared vision has a positive effect upon an increased level of participation in leadership activities*	H1: Supported
*H2: Compassion has a positive effect upon an increased level of participation in leadership activities*	H2: Supported
*H3: Mood has a positive effect upon an increased level of participation in leadership activities*	H3: Not Supported
*H4: Vision partially mediates the positive relationship of role endorsement upon participation, such that the effect is stronger*	H4: Supported
*H5: Compassion partially mediates the positive relationship of role endorsement upon participation, such that the effect is stronger*	H5: Supported
*H6: Mood partially mediates the positive relationship of role endorsement upon participation*	H6: Not Supported
*H7: Vision partially and positively mediates the negative relationship of role conflict upon participation*	H7: Vision fully mediates the relationship between role conflict and participation
*H8: Compassion partially mediates the negative relationship of role conflict upon participation*	H8: Compassion fully mediates the relationship between role conflict and participation
*H9: Mood partially mediates the negative relationship of role conflict upon participation so that the relationship of compassion to participation is positive*	H9: Not Supported

**Table 6:** **PLS-CFA measurement model results**.

**Construct**	**Loadings or weight**	**Standard error**	***t*-statistic**	**Composite reliability**	**Average variance extracted**
Role conflict				0.831	0.553
RC1	0.8103	0.0238	15.4711		
RC2	0.8082	0.0276	14.451		
RC3	0.6396	0.0302	7.979		
RC4	0.7028	0.0282	11.35		
Role endorsement				0.907	0.663
RE2	0.6799	0.015	13.871		
RE3	0.8816	0.0089	29.3311		
RE4	0.7718	0.0103	20.4912		
RE5	0.8897	0.0090	31.4706		
RE6	0.8295	0.0096	26.5433		
Vision				0.929	0.621
VIS1	0.7027	0.0093	15.5335		
VIS2	0.7028	0.0082	16.5069		
VIS3	0.7931	0.0073	24.6386		
VIS4	0.8114	0.0068	23.5194		
VIS5	0.7949	0.0084	19.6612		
VIS6	0.8413	0.0064	26.8095		
VIS7	0.8111	0.0081	19.0003		
VIS8	0.8346	0.0075	21.0442		
Compassion				0.884	0.562
COMP1	0.7927	0.0124	18.4857		
COMP2	0.7182	0.0153	13.8153		
COMP3	0.7049	0.0139	15.7524		
COMP4	0.7980	0.0131	17.8802		
COMP4	0.6577	0.0190	9.0571		
COMP6	0.8127	0.0125	20.8252		
Participation					
PART1	0.5394	0.1217	4.4338		
PART2	0.6406	0.1264	5.0676		
PART3	0.0786	0.1233	0.6375		
PART4	0.0713	0.1094	0.6518		

*Participation is a formative construct and values shown represent item weights*.

The final model, shown in Figure 4, shows the relationships and significant paths between constructs, as well as the *R*^2^-values for each construct. The details of the structural model can be found in the Appendix.

The results structural model testing provide evidence to support H1 and H2, vision (β = 0.149^*^) and compassion (β = 0.210^***^), have a significant and positive relationship with participation. Vision plays a mediating role in the relationship of role conflict and role endorsement to participation. The direct effect of role endorsement to participation was significant, as was the indirect effect via both vision and compassion. Moreover, role endorsement showed a significant direct effect with the vision (β = 0.572^***^) and compassion (β = 0.495^***^) mediators, and both vision (β = 0.149^*^) and compassion (β = 0.149^*^) had significant relationships with participation. These findings are therefore consistent with the hypotheses of partially mediated effects; therefore H4 and H5 are both supported.

It was hypothesized that the role conflict on participation would be partially mediated by both vision and compassion. Instead, it was found that the there was not a significant relationship from role conflict to participation, and role conflict showed a significant negative direct effect with the vision (β = −0.186^***^) and compassion (β = −0.177^***^) mediators, and as previously stated, both vision (β = 0.149^*^) and compassion (β = 0.149^*^) had significant relationships with participation. In summary, the results of indicate that role conflict has a direct effect on the mediators of vision and compassion, and both of these mediators has a significant relationship with participation, and that the direct effect of role conflict to participation is no longer significant. These results are consistent with the hypothesis of a full mediation for H7 and H8.

H4, H6, and H9 were not supported, as compassion, a sub-construct of the P/NEA Scale was not found to be significant.

## Discussion

Insights were applied from positive psychology, social psychology and management literature to demonstrate that role factors, such as role conflict and role endorsement, are an important consideration in participation by physician leaders. Specifically, this study found support that role conflict negatively affects participation; while role endorsement has a positive relationship with participation.

These results also show support for the argument that positivity, in this instance vision and compassion, mediate the relationship of role factors and participation. The largest effect found in this model was the relationship of role endorsement to vision, which may speak to the importance of an individual being endorsed in their role by both their peers and organization, in addition to their own certainty in their authority. This is inline with DeRue and Ashford's (2010) theory on the relational construction of identity. These findings show that including vision and compassion into the physician leadership framework, there is a noteworthy impact upon participation, which may precede effectiveness.

The findings also confirm the importance of the leader-member relationship, as it relates to the importance of the endorsement of the leadership role by peers. Specifically, the mediators of vision and compassion partially mediate the relationship of role endorsement to participation.

In testing the mediated relationship of role conflict and participation, surprisingly, full mediation was found in that that the association was completely accounted for by vision and compassion (James et al., 2006). This finding reinforces the importance of positive affect as a mediating factor for physician leaders. Boyatzis suggests that leadership development occurs in an iterative cycle of “discontinuities,” which results in desired change (2008). This process is described by his intentional change theory (ICT) (Boyatzis, 2008). The shared vision of the ideal self, in this case the embracement of the secondary identity of leader by a physician, their peers and the organization in which they function, produces the desired change. These changes can occur not only at the individual level, but also at the didactic, group or organizational level, etc. (Boyatzis, 2008), therefore the opportunity exists for the healthcare organization to produce desired change that will result in the a shared vision at the group level. This shared vision is what allows for an increase in participation, and potentially effectiveness, and offers a mediating role in positively impacting the relationship of role factors, both positive and negative, to outcomes.

In the case of role conflict, the findings demonstrate the significant role that vision and compassion play within the model, as these mediating factors fully explain the relationship to participation. These results illuminate the importance of positivity in buffering role conflict, with the hope of increasing participation and potentially effectiveness. It was unexpected, however, that no significant differences were found in the model when testing for part time physician leaders vs. full time physician leaders. It was anticipated that there may be a difference in these results involving role conflict, as a previous inquiry by this author suggested that there was a distinct difference between how part and full time leaders viewed their role as it pertains to conflict. Role endorsement was also found to be an important factor in this study. DeRue and Ashford (2010) propositioned that leadership is a mutual influence process among individuals, expressly a socially constructed and reciprocal relationship between leaders and followers that is co-created and mutually reinforced.

Finally, it is curious that mood was not found to be significant in our testing, while the other two sub-scales of the P/NEA were found to be significant. It may be that mood is something physicians do not have the luxury of allowing to impact their work in life and death situations, therefore they have conditioned themselves not to allow affect from mood. With enough conditioning and time, this mood may not have a significant impact upon on a physician's behavior, whether they are acting in a clinical capacity or not.

Further research is needed to explore additional mediating factors, which may explain the relationship of role factors to participation.

## Limitations

A potential limitation to this study is one that may actually strengthen the results- the fact that the sample is comprised of both part and full time physician leaders who have *self-selected* to join the ACPE due to their interest in bettering themselves as leaders. As such, these results may be even more important for healthcare leaders to consider, as even those physicians who are committed to leadership still may struggle with role factors that impact their participation. Although one may find issue with whether or not these results are representative of the entire physician leader population, the significance of the relationships in the model speak to the importance of role related factors and positivity for participation of physician leaders. The sample also is diverse with regard to roles and organization size and type, which may offer a more robust interpretation of the findings; however, a more focused study would have the ability to better analyze the impact of the organizational climate across the sample.

While great strides were taken to protect the results from common methods bias, no statistical test can guarantee such bias does not exist within these results (Podsakoff et al., 2003). If possible, an evenly distributed sample by role would be preferable, which may have led to further insight. An attempt was made at collection of 360° data; however, the responding sample of data from secondary respondents was too low to include in the results. Finally, the dependent variable, participation, could have been measured on a different scale rather a psychometric scale, which may have affected the results.

## Implications for practice and future research

As the first study to empirically examine the impact of relational and organizational endorsement of role, this study offers previously undiscovered insight as to the impact role perception to healthcare leaders concerned with physician leadership.

### Practice

A practical implication of these findings is the understanding of the factors that influence the acceptance of a leadership identity for physicians by healthcare administrators, so that they may positively influence the interpreted psychological climate by physician leaders. If healthcare leaders know the factors that influence physician leaders to fully accept and engage in their role, they will be better prepared to assist in the development of physician leaders. Pratt et al. (2006) found that “achieving alignment between identity and work is a fundamental motivator in identity construction” (2006, p. 255).

While psychological climate is an understanding of meaning by the individual, there are several ways in which an organization can influence that perception. At the organizational level, healthcare leaders may be informed by the impact of vision and compassion upon a physician leader's engagement and increase awareness surrounding these concepts. Leadership development workshops and programs can aid in an individual's self- awareness and an understanding of the factors that enable them to participate at a higher level and potentially become a better leader.

Physicians entering into leadership roles may also be informed by these findings. If physicians are aware of the factors that may limit or enhance how they enact their role as a leader, they may be better prepared to deal with the challenges. The basic realization that they may struggle with the acceptance of the secondary identity as a leader may alone be enough to encourage them to explore options to overcome the limitations to acceptance of that role.

Finally, these results should also inform medical school administrators and faculty members of the importance of including leadership skills and specifically emotional and social competencies into the curriculum. Chaudry et al. note “because leadership skill sets are not emphasized during training and practice, physicians, whose education is rooted in quantitative science, tend to address most problems with technical solutions” (Chaudry et al., 2008, p. 219).

Stoller et al. (2013) have suggested a curriculum to develop self-awareness in physicians, which begins as a medical student and develops the individual as they move from student, to physician, to member of a healthcare team and finally to a leadership role. I propose that this would greatly benefit a physician as they move through their career and into leadership roles; not only in developing their own emotional intelligence, but also to guide them in the process of adoption of a leadership identity and endorsement of their role.

### Future research

Future research should continue to examine the impact of role endorsement upon not only organizational participation, but also effectiveness. As well, although no significant differences were found between those in part and full time leadership roles within this study, this may be an aspect for further examination, especially with regard to individuals from a single organization.

It was anticipated going into this exploration that there may be a difference in the results involving role conflict for part and full time physician leaders; however, there were no significant differences found related to part or full time status. Therefore, a more detailed exploration of how part or full time status may be impacted by organizational climate may be beneficial.

This model only examined the linear relationships associated with the intervening effects. However, moderator relationships could be incorporated into future explorations involving these constructs.

### Conflict of interest statement

The author declares that the research was conducted in the absence of any commercial or financial relationships that could be construed as a potential conflict of interest.
