# Supplementary material for: The affect of vision and compassion upon role factors in physician leadership
Source: Front Psychol. 2015 May 8;6:442. doi: 10.3389/fpsyg.2015.00442 (PMC4424809; doi:10.3389/fpsyg.2015.00442)
Supplement: Supplementary file 1 [file DataSheet1.DOCX]

# APPENDIX A: Summary of Construct Operationalization

| **Construct** | **Definition** | **Indicators** |
| --- | --- | --- |
| **Role Endorsement**  **(reflective)** | Whether an individual is recognized by their peers and organization as having the authority to carry out that role. | 1. I feel that I am a leader. 2. My peers acknowledge my authority. 3. I have the authority that I need to carry out my role. 4. I feel my leadership role is the same as what my title states. 5. I feel that the organization provides me with the authority to carry out my (leadership) role. 6. My leadership role is recognized throughout the organization. |
| **Positive Relationships**  **(P/NEA)**  **(reflective)** | The positive emotional attractor (PEA) triggers constructive cognitive and physiological responses that enhance an individual's openness and adaptive behaviors. The negative emotional attractor (NEA) triggers conversely may compromise an individual's effectiveness.  There are three sub-constructs of PNEA, vision, compassion and overall positive mood. | **Vision**   1. Management emphasizes a vision for the future. 2. We often discuss possibilities for the future 3. Our future as an organization will be better than our past. 4. I feel inspired by our vision and mission. 5. We are encouraged by management to and build on our strengths. 6. Our work is focused on our vision or mission. 7. Our purpose as an organization is clear in our vision or mission. 8. Management emphasizes our current strengths.   **Compassion**   1. I do not feel trusted by my colleagues. * 2. I feel trusted by my colleagues. 3. I care about my colleagues at work. 4. I do not trust my colleagues. * 5. I do not care about my colleagues at work. * 6. I trust my colleagues.   **Overall Positive Mood**   1. This is a great place to work. 2. I enjoy working here. 3. I do not like working here. * 4. Working here is a joy. 5. If I had a choice, I would work somewhere else. * 6. Overall, it feels good to work here.   ** Note that the questions with an asterisk following are reverse coded.* |
| **Role Conflict**  **(reflective)** | Incompatibility of requirements within the role. | 1. I have to “feel my way” in performing my duties. 2. [There is a] lack of policies and guidelines to help me. 3. I work with two or more groups who operate quite differently. 4. I do things that are apt to be accepted by one person and not accepted by others. |
| **Participation**  **(formative)** |  | 1. Frequently makes [innovative] suggestions to coworkers. [Innovative replaced the original word, creative.] 2. Encourages others to speak up at meetings 3. Keeps well informed where opinion might benefit the organization. 4. Does not pursue additional training to improve performance.* 5. Does not push [peers and] superiors to perform to higher standards. [“Peers and” added to the original question.]*   ** Note that the questions with an asterisk following are reverse coded.* |
| **Controls** | Age  Gender  Current Position Tenure  Organization Tenure  Part/Full Time Leadership Role | 1. Age: How old are you? 2. Gender: What is your gender? 3. Current position tenure: How long have you been in your current leadership position? 4. Organization tenure: How long have you been with the organization? 5. Part/Full Time Leadership Role: Do you hold a part time or full time leadership role within your organization?   Do you receive compensation for a part time leadership role within your organization? |

# APPENDIX B: EFA

| **KMO and Bartlett's Test** | | |
| --- | --- | --- |
| Kaiser-Meyer-Olkin Measure of Sampling Adequacy. | | .932 |
| Bartlett's Test of Sphericity | Approx. Chi-Square | 8935.759 |
|  | df | 378 |
|  | Sig. | .000 |

| **Communalities** | | |
| --- | --- | --- |
|  | Initial | Extraction |
| COMP1 | .509 | .577 |
| COMP2 | .417 | .428 |
| COMP3 | .514 | .445 |
| COMP4 | .581 | .621 |
| COMP5 | .440 | .366 |
| COMP6 | .604 | .621 |
| OC1 | .170 | .208 |
| OC2 | .209 | .270 |
| OC3 | .221 | .311 |
| OC4 | .100 | .107 |
| OC5 | .140 | .130 |
| RC1 | .459 | .542 |
| RC2 | .441 | .488 |
| RC3 | .289 | .331 |
| RC4 | .319 | .353 |
| ROE2 | .412 | .418 |
| ROE3 | .723 | .757 |
| ROE4 | .486 | .507 |
| ROE5 | .764 | .816 |
| ROE6 | .565 | .610 |
| VIS1 | .450 | .444 |
| VIS2 | .440 | .440 |
| VIS3 | .573 | .566 |
| VIS4 | .611 | .630 |
| VIS5 | .558 | .566 |
| VIS6 | .651 | .684 |
| VIS7 | .630 | .643 |
| VIS8 | .630 | .678 |
| Extraction Method: Principal Axis Factoring. | | |

| **Pattern Matrix^a^** | | | | | |
| --- | --- | --- | --- | --- | --- |
|  | Factor | | | | |
|  | 1 | 2 | 3 | 4 | 5 |
| VIS8 | .876 |  |  |  |  |
| VIS7 | .853 |  |  |  |  |
| VIS6 | .818 |  |  |  |  |
| VIS4 | .805 |  |  |  |  |
| VIS5 | .689 |  |  |  |  |
| VIS3 | .604 |  |  |  |  |
| VIS1 | .590 |  |  |  |  |
| VIS2 | .586 |  |  |  |  |
| ROE5 |  | .866 |  |  |  |
| ROE3 |  | .854 |  |  |  |
| ROE4 |  | .721 |  |  |  |
| ROE6 |  | .695 |  |  |  |
| ROE2 |  | .464 |  |  | .252 |
| COMP1 |  |  | .773 |  |  |
| COMP4 |  |  | .767 |  |  |
| COMP6 |  |  | .656 |  |  |
| COMP2 |  |  | .620 |  |  |
| COMP5 |  |  | .518 |  | .207 |
| COMP3 | .218 |  | .437 |  | .232 |
| RC1 |  |  |  | -.736 |  |
| RC2 |  |  |  | -.652 |  |
| RC3 |  |  |  | -.591 |  |
| RC4 |  |  |  | -.541 |  |
| OC3 |  |  |  |  | .532 |
| OC2 |  |  |  |  | .476 |
| OC1 |  |  |  |  | .467 |
| OC4 |  |  |  |  | .352 |
| OC5 |  |  |  |  | .294 |
| Extraction Method: Principal Axis Factoring.  Rotation Method: Promax with Kaiser Normalization. | | | | | |
| a. Rotation converged in 7 iterations. | | | | | |
|  | | | | | |


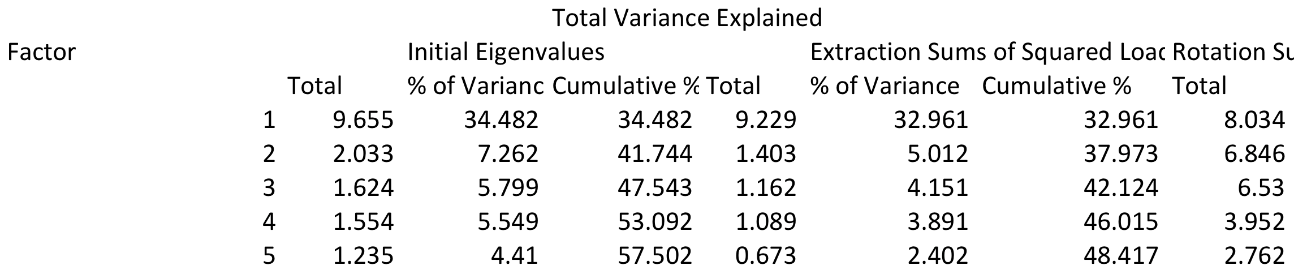


Role Conflict

| **Reliability Statistics** | |
| --- | --- |
| Cronbach's Alpha | N of Items |
| .728 | 4 |

Role Endorsement

| **Reliability Statistics** | |
| --- | --- |
| Cronbach's Alpha | N of Items |
| .872 | 5 |

Vision

| **Reliability Statistics** | |
| --- | --- |
| Cronbach's Alpha | N of Items |
| .912 | 8 |

Compassion

| **Reliability Statistics** | |
| --- | --- |
| Cronbach's Alpha | N of Items |
| .841 | 6 |
